# Clinical characteristics and management of paroxysmal nocturnal hemoglobinuria in the Middle East: a narrative review

**DOI:** 10.1007/s10238-025-01834-5

**Published:** 2025-08-31

**Authors:** Mohammed Almakadi, Noura AlHashim, Murtadha Al-Khabori, Hazzaa Alzahrani, Hani Yousif Osman, Mervat Mattar, Ahmed Sabah

**Affiliations:** 1 Section of Hematology, Department of Medicine, King Faisal Specialist Hospital & Research Center, Madinah, Saudi Arabia; 2 Mubarak Alkabeer Hospital, Ministry of Health, Kuwait, Kuwait; 3 College of Medicine and Health Sciences, Sultan Qaboos University, Muscat, Oman; 4 National Hematology and Bone Marrow Transplant Center, University Medical City, Muscat, Oman; 5 Hematology, Stem Cell Transplant & Cellular Therapy Department, King Faisal Specialist Hospital & Research Center, Riyadh, Saudi Arabia; 6 Tawam Hospital, Alain, United Arab Emirates; 7 Clinical Hematology Unit, Internal Medicine Department, Cairo University, Giza, Egypt; 8 Hematology Department, Kadhimiya Hematology Center, Baghdad, Iraq

**Keywords:** Paroxysmal nocturnal hemoglobinuria, Eculizumab, C5 inhibitors, Middle East

## Abstract

Paroxysmal nocturnal hemoglobinuria (PNH) is a rare hematological disorder caused by uncontrolled terminal complement activation of blood cells. It is associated with intravascular hemolysis, thromboembolic events, organ damage, impaired quality of life and premature mortality. As there are no PNH registry data from Middle Eastern countries, little is known about its management in the region. This narrative review summarizes available data on the prevalence, characteristics, diagnosis and treatment of PNH in Middle Eastern populations of Arabic origin. A search of PubMed and EMBASE from inception to 31 May 2025 identified 15 relevant publications: five from Saudi Arabia, four from Iran, two from Kuwait, one each from Egypt, Oman, Lebanon, and one reporting a Middle Eastern patient treated in Germany. The estimated incidence rate of PNH in an Omani cohort was 1.9 per 5 million population. Mean and median ages at PNH diagnosis were 38 years (Iranian retrospective review, *n* = 81) and 22.5 years (Omani case series, *n* = 10), respectively. Where reported, anemia and fatigue were common presenting symptoms. Few publications reported on treatment with C5 inhibitors, although available data indicate that eculizumab generally improves patients’ clinical condition. Uptake and clinical use of ravulizumab in the Middle East remains undocumented. Subject to limitations of the available data, the management approach to PNH in the Middle East appears to be generally consistent with that reported in other regions. However, additional data are required to gain greater insight into the status of PNH and its management in Middle Eastern populations.

## Introduction

Paroxysmal nocturnal hemoglobinuria (PNH) is a rare, chronic, potentially life-threatening hematological disorder caused by uncontrolled terminal complement activation of blood cells. The condition is associated with intravascular hemolysis, thromboembolic events, organ damage, impaired quality of life and premature mortality [1–3]. Recognized as a disease entity for more than 100 years [2–4], PNH is extensively documented in the clinical literature, with the number of publications increasing dramatically in recent years coinciding with the introduction of complement-targeted therapeutics. Since its establishment in 2004, the International PNH Registry (NCT01374360) has collected and reported disease and treatment-related information about PNH [5–9]. However, because not all countries participate in the International PNH Registry, little is known about the epidemiology and management of PNH in certain world regions. Against this background, and after a brief overview of the disorder, this narrative review summarizes available data on the prevalence, characteristics, diagnosis, and treatment of PNH in the Middle East.

## Overview of paroxysmal nocturnal hemoglobinuria

### Epidemiology

Due to the rarity of PNH, its true incidence is largely unknown. A prevalence of 12–13 cases per 1 million people was reported in the USA [10], and an incidence rate of 0.35 cases per 100,000 person-years in the UK [11]. PNH can occur in any sex, race, geographical region or age group, although median age at onset is 30–40 years [5, 8] and pediatric cases account for less than 15% of reported cases [12, 13]. There is some indication that the clinical manifestations of PNH may vary by ethnicity [1, 3, 4], although the most recent analysis of the International PNH Registry (78.4% white, 16.3% Asian, 3.0% black, 2.3% other) did not conduct comparisons across racial and/or ethnic groups [8].

### Pathogenesis

PNH is a terminal complement disorder caused primarily by acquired somatic mutations in the X-linked phosphatidylinositol glycan class A (*PIGA*) gene of bone marrow stem cells [1–3, 14]. All blood cell lineages are affected, including red blood cells, leucocytes, and platelets [15]. Mutations in *PIGA* result in a deficiency of the glycosyl phosphatidylinositol (GPI)-anchored complement regulatory proteins CD55 and CD59 [1–3]. Blood cells become susceptible to complement-mediated attack, resulting in intravascular red blood cell lysis, and a vasoconstrictive and procoagulant state [2, 3, 16–18]. PNH often occurs in the context of bone marrow failure disorders, particularly aplastic anemia [2–4].

### Diagnosis and classification

Minimal essential criteria required to diagnose and categorize PNH have been published [1]. One of the main elements is high sensitivity flow cytometric analysis of peripheral blood erythrocytes, granulocytes, or both using primary antibodies against GPI-anchored proteins or a fluorescein-labeled proaerolysin (FLAER) assay to reveal hematopoietic cells deficient in GPI-anchored proteins.

PNH is classified into three main subtypes: classical hemolytic PNH with evidence of intravascular hemolysis; PNH (with hemolysis) in association with a defined bone marrow disorder; and subclinical (asymptomatic) PNH with small clones but no hemolysis [1]. However, not all patients fit into one of these categories [2].

### Clinical characteristics

PNH is characterized by intravascular hemolysis, thromboembolism, and organ damage [2, 3]. Clinical manifestations are variable and include anemia, thrombosis, smooth muscle dystonia (which can cause abdominal pain and dysphagia), fatigue, hemoglobinuria, renal impairment, dyspnea, and pulmonary hypertension [2, 3, 8, 19]. Venous thrombosis is common and, without treatment of the underlying PNH, can result in substantial morbidity and mortality [2, 3]. Arterial thrombosis is a less common but serious complication of PNH as sites include the cerebral, cardiac, and mesenteric arteries [4, 15]. Elevated lactate dehydrogenase concentrations (≥ 1.5 times the upper limit of normal), the biomarker of intravascular hemolysis, are associated with a significantly increased risk of thromboembolism and mortality [20–22].

As many of the symptoms of PNH are non-specific, the diagnosis is frequently missed or delayed [23]. Screening for PNH is recommended for high-risk patients, such as those with unexplained chronic intravascular hemolysis, unexplained hemoglobinuria, thrombosis at unusual sites (e.g., Budd-Chiari syndrome or cerebral thrombosis), aplastic anemia or myelodysplastic syndrome, and young patients with thrombosis in combination with hemolytic anemia or cytopenia [1, 24].

### Treatment

**Fig. 1:**
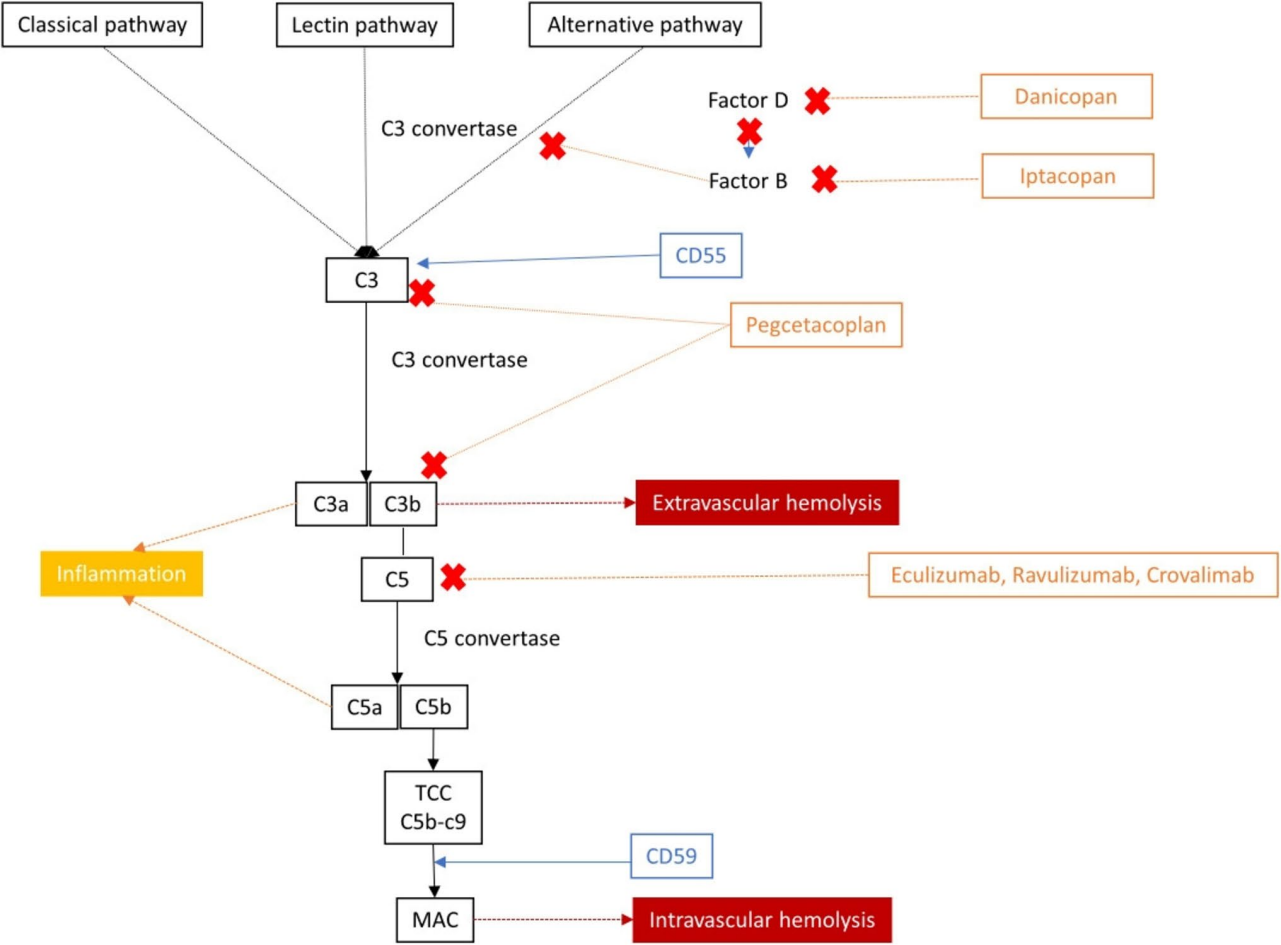
Terminal complement cascade relevant to paroxysmal nocturnal hemoglobinuria and targets of inhibition in the complement pathway. C3, complement component 3; C5, complement component 5; TCC, terminal complement complex; MAC, membrane attack complex

Complement inhibition is central to the current pharmacological management of PNH (Fig. 1). Eculizumab is a humanized monoclonal antibody that binds to complement protein C5 [25]. By reducing intravascular hemolysis and transfusion rates, and prolonging survival, eculizumab has yielded substantial improvements in PNH outcomes [26, 27]. The second-generation C5 inhibitor ravulizumab has an improved dosing schedule (every 8 weeks), provides complete terminal complement inhibition, and has a safety profile similar to that of eculizumab [28–30]. Ravulizumab is increasingly used because of its more convenient dosing schedule and favorable breakthrough-hemolysis profile seen in phase 3 trials [28–31].

Pegcetacoplan, a C3 complement inhibitor that can potentially prevent both intravascular and extravascular hemolysis [32, 33], has been shown to be efficacious in treating PNH with clinically significant extravascular hemolysis, but there is a risk of severe breakthrough intravascular hemolysis [34, 35]. C3 inhibition is recommended for certain PNH patients only [36, 37]. Other novel treatment options are also now available [38–42]. At present, C5 inhibitors are the preferred front-line approach to treating PNH owing to their proven efficacy and longer-term use experience [37].

Supportive measures for PNH generally include blood transfusion, antithrombosis prophylaxis, and antibiotic treatment [3, 25]. Allogeneic hematopoietic stem cell transplantation has a limited role in PNH patients, as it carries a high risk of mortality, and is best reserved for severely ill patients with bone marrow failure [3, 43].

## Management of PNH in the Middle East

### Systematic literature search methodology and results

A bibliographic search was undertaken of the PubMed and EMBASE databases from inception to 31 May 2025 to identify relevant studies on PNH in Middle Eastern populations of Arabic origin (excluding Israel and Turkey). The language was limited to English. The search strategy was adapted for each database and was built by combining MESH/EMTREE terms (PubMed and EMBASE controlled vocabulary) and free-text searching in the title/abstract fields. Search terms and their variations were identified based on main topics, keywords and controlled vocabularies. To ensure the comprehensiveness of the search, term variations were included in the free-text search, and truncation was used for some terms.

The PubMed search strategy was as follows. Search #1: (((((“hemoglobinuria, paroxysmal”[MeSH Terms] AND nocturna*[Title/Abstract]) OR (paroxysmal hemoglobinuria nocturna*[Title/Abstract])) OR (paroxysmal nocturna* hemoglobinuria[Title/Abstract])) OR (paroxysmal nocturna* haemoglobinuria[Title/Abstract])) OR (marchiafava micheli syndrom*[Title/Abstract])) OR (marchiafava-micheli syndrom*[Title/Abstract]). Search #2: “Pan Arab”[Title/Abstract: ~ 1] OR “Arabian Gulf”[Title/Abstract: ~ 1] OR “Gulf Cooperation Council”[Title/Abstract: ~ 1] OR “Middle-East”[Title/Abstract] OR “Middle-East”[Title/Abstract] OR “middle east*”[Title/Abstract] OR “Akrotiri Dhekelia”[Title/Abstract: ~ 1] OR “Episkopi”[Title/Abstract] OR “Bahrain”[Title/Abstract] OR “Manama”[Title/Abstract] OR “bahraini*”[Title/Abstract] OR “Egypt”[Title/Abstract] OR “Cairo”[Title/Abstract] OR “egyptian*”[Title/Abstract] OR “Iran”[Title/Abstract] OR “Tehran”[Title/Abstract] OR “iranian*”[Title/Abstract] OR “Iraq”[Title/Abstract] OR “Baghdad”[Title/Abstract] OR “iraqi*”[Title/Abstract] OR “Jordan”[Title/Abstract] OR “Amman”[Title/Abstract] OR “jordanian*”[Title/Abstract] OR “Kuwait”[Title/Abstract] OR “kuwaiti”[Title/Abstract] OR “Lebanon”[Title/Abstract] OR “Beirut”[Title/Abstract] OR “Oman”[Title/Abstract] OR “Muscat”[Title/Abstract] OR “omani*”[Title/Abstract] OR “Palestine”[Title/Abstract] OR “Ramallah”[Title/Abstract] OR “palestinian*”[Title/Abstract] OR “Qatar”[Title/Abstract] OR “Doha”[Title/Abstract] OR “qatari*”[Title/Abstract] OR “saudi arabia”[Title/Abstract] OR “Riyadh”[Title/Abstract] OR “saudi*”[Title/Abstract] OR “Syria”[Title/Abstract] OR “Damascus”[Title/Abstract] OR “syrian*”[Title/Abstract] OR “united arab emirates”[Title/Abstract] OR “abu dahbi”[Title/Abstract] OR “emirati*”[Title/Abstract] OR “Yemen”[Title/Abstract] OR “Sanaa”[Title/Abstract] OR “Sana’a”[Title/Abstract] OR “Aden”[Title/Abstract] OR “yemeni*”[Title/Abstract] OR “yemenite*”[Title/Abstract]. Search #3: #1 AND #2.

The EMBASE search strategy was as follows: 1. “paroxysmal hemoglobinuria nocturna*”.ab,ti.; 2. “paroxysmal nocturna* hemoglobinuria”.ab,ti.; 3. paroxysmal hemoglobinuria/4. “nocturna*”.ab,ti.; 5. 3 and 4; 6. (paroxysmal hemoglobinuria adj2 “nocturna*”).ab,ti.; 7. “paroxysmal nocturna* haemoglobinuria”.ab,ti.; 8. Marchiafava-Micheli syndrom*.ab,ti.; 9. 1 or 2 or 5 or 6 or 7 or 8; 10. (Pan adj1 Arab).ab,ti.; 11. (Arabian adj1 Gulf).ab,ti.; 12. Gulf Cooperation Council.ab,ti.; 13. (Middle East or Middle-East or Middle East* or “Akrotiri and Dhekelia” or Episkopi or Bahrain or Manama or bahraini* or Egypt or Cairo or egyptian* or Iran or Tehran or iranian* or Iraq or Baghdad or iraqi* or Jordan or Amman or jordanian* or Kuwait or kuwaiti or Lebanon or Beirut or belanese* or Oman or Muscat or omani* or Palestine or Ramallah or palestinian* or Qatar or Doha or qatari* or Saudi Arabia or Riyadh or saudi* or Syria or Damascus or syrian* or United Arab Emirates or Abu Dahbi or emirati* or Yemen or Sanaa or Sana’a or Aden or yemeni* or yemenite*).ab,ti.; 14. 10 or 11 or 12 or 13; 15. 9 and 14; 16. limit 15 to English language.

**Fig. 2:**
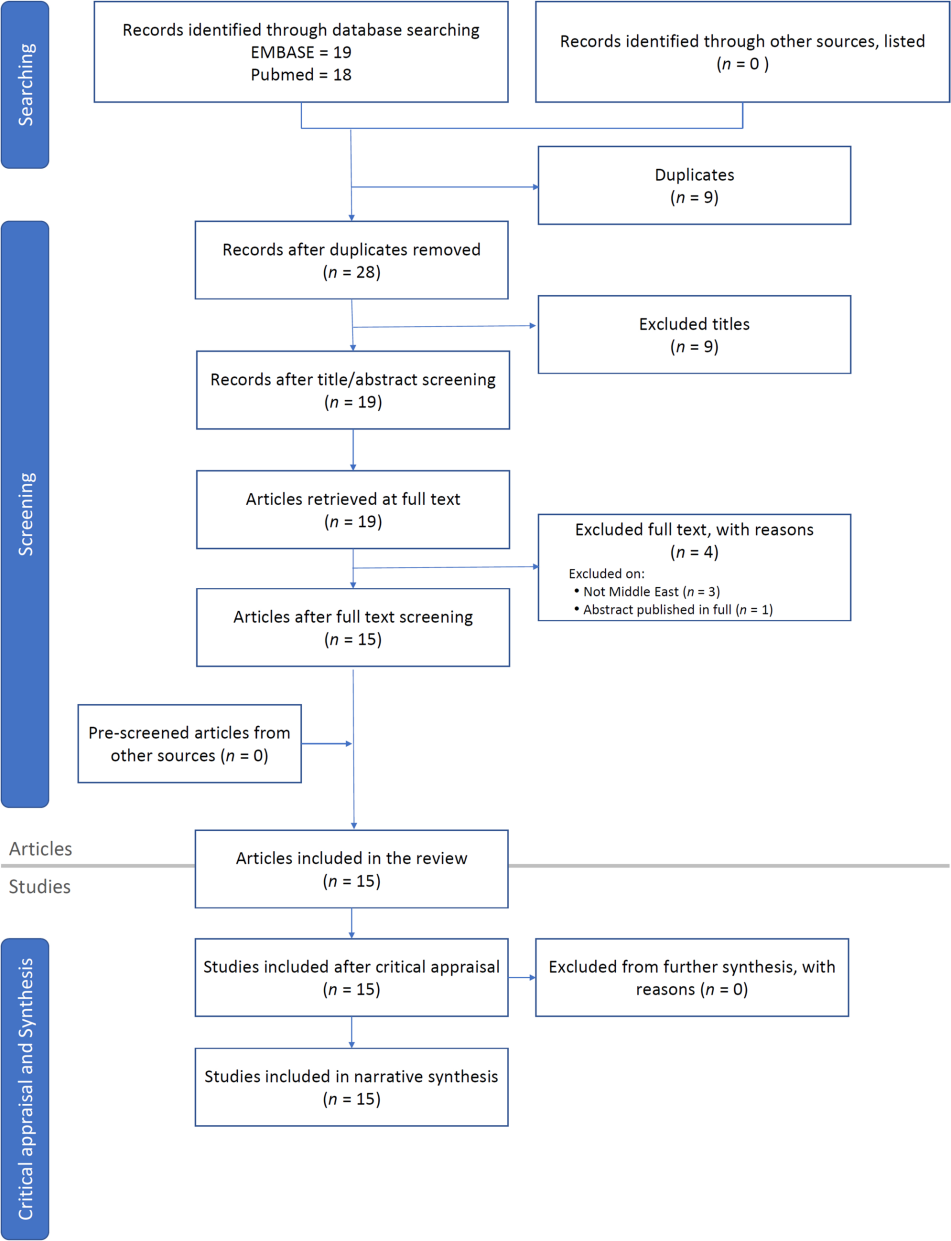
Literature search flowchart

The database searches retrieved 37 records (Fig. 2). After duplicates were removed, a preliminary manual screening based on titles/abstracts was conducted by the medical writers; nonrelevant articles were excluded. Of 19 full-text articles retrieved, four were excluded: three articles were not specific to the Middle East region, and one was an abstract subsequently published as a full article. Reference lists of relevant articles were searched for any additional publications. All available studies, and case series/reports involving patients with PNH from Middle Eastern countries were included in the review. The 15 publications comprise seven studies/case series [44–50] and eight case reports [51–58] (Table 1). Publication dates range from 1998 to 2022. About half of included articles (*n* = 8) were published before 2016.

**Table 1:** Summary of available literature reporting on patients with paroxysmal nocturnal hemoglobinuria in the Middle East

Author	Country	Design	Year(s)	N	Population	Main findings
Rizk et al. [44]	Egypt	Case series	2002	11	Pediatric aplastic anemia	PNH clones detected (using IHC/CD59) in 4/11 (all aged > 6 years)
AlGhasham et al. [45]	Saudi Arabia	Case series	2012–2013	11	Patients with indication for PNH screening	14/366 (4%) samples submitted for PNH screening were positive. Among 11 evaluable patients (age 18–50 years), 8 (73%) presented with aplastic anemia. Median clone size 15% (range 0.7–56%) in RBC [CD59] and 63% (3.8–100%) in granulocytes and monocytes [FLAER/CD24]
Kamranzadeh Fumani et al. [46]	Iran	Case series	2002–2014	13	PNH patients undergoing HSCT	All had full-matched sibling donors. Mean age at transplant 27.5 years (range 18–47); mean time from diagnosis to transplant 41.3 months (range 5–132). Mortality rate 23% (*n* = 3); all from graft-versus-host disease. Survival rate 74% at both 1 year and 13 years
Al-Dosari et al. [47]	Saudi Arabia	Case series	2013–2021	4	Pregnant PNH patients	Ten pregnancies in four women (three treated with eculizumab). Antepartum symptoms ranged from mild (e.g., epistaxis) to life-threatening thrombosis. Transfusions required during four pregnancies. Pregnancy outcomes varied: three healthy infants; one infant with congenital anomaly; five IUFD/spontaneous abortion; one pregnancy ongoing at time of reporting. One maternal thrombosis in postpartum period
AlMozain et al. [48]	Saudi Arabia	Retrospective study	2009–2016	183	Patients evaluated for MDS	Among 155 adults evaluated for possible MDS, 4% were found to have PNH
Al-Riyami et al. [49]	Oman	Case series	2012–2019	10	PNH patients	Estimated incidence 1.9 cases/5 million. Median age 22.5 years (range 16–49). Clinical manifestations included fatigue (80%) and anemia (70%). Six patients had hemolytic anemia. Median clone size: neutrophils 95.5% (FLAER/CD24); monocytes 91.6% (FLAER/CD14). Treatment: eculizumab (*n* = 5), immunosuppressants (*n* = 3), bone marrow transplant (*n* = 4). During median follow-up of 62 months, one thrombosis and no deaths occurred
Jahangirpour et al. [50]	Iran	Case series	2014–2019	81	PNH patients	Mean age 38 years (range 11–79); 53.1% male. Symptoms/signs included fatigue, jaundice, hematuria; 91.4% had anemia, 74.1% had thrombocytopenia and 51.9% had neutropenia. Average clone sizes: RBC 29.6% (range 0.34–87.8%; CD235a/59/55); monocytes 58.9% (range 1.59–99%; FLAER/CD45/15/24), granulocytes 58.9% (range 0.07–98.44%; FLAER/CD45/64/14). Higher average PNH clone size in age group 25–45 years versus other age groups
Al-Harbi et al. [51]	Saudi Arabia	Case reports	1987, 1993	2	PNH and acute renal failure	Patient 1 (male, 33 years) diagnosed with PNH at same time as acute renal failure. Patient 2 (male, 30 years) developed acute renal failure 5 years after PNH diagnosis (with pre-existing aplastic anemia) and was receiving immunoglobulin and danazol. Renal function recovered in both patients after supportive therapy, including hemodialysis; fully normal after 2 or 6 weeks
Mooraki et al. [52]	Iran	Case report	1998	1	PNH and acute renal failure	Diagnostic work-up of young male with acute renal failure requiring hemodialysis led to diagnosis of PNH. Renal function recovered fully after 25 days
Khajehdehi et al. [53]	Iran	Case report	1999	1	PNH and acute renal failure	39-year-old man with known PNH developed acute renal failure with gross hematuria and prolonged oliguria (8 weeks) in the absence of a concurrent aggravating factor. Treatment included hemodialysis. Full recovery of renal function after 10 weeks
Gupta et al. [54]	Kuwait	Case report	2007	1	PNH in SLE patient	60-year-old woman with SLE developed portal vein thrombosis and concurrent severe thrombocytopenia. Investigations showed patient had developed PNH
Al-Jafar et al. [55]	Kuwait	Case report	2015	1	High thrombogenic risk PNH	Adult woman with Budd-Chiari syndrome (2006), portal vein stent and receiving anticoagulants for ongoing high risk of thrombosis was diagnosed with PNH in 2007. Eculizumab was started in 2011, at which time she refused any further anticoagulant therapy. No thrombotic events have occurred in the 4 years since then
Boqari et al. [56]	Saudi Arabia	Case report	2015	1	PNH and herb-induced bone marrow and renal damage	48-year-old woman presented with anemia and acute kidney injury after taking herbal medicine of unknown constituents. During work-up she was diagnosed with PNH. Also with renal hemosiderosis, herb-induced acute interstitial nephritis and acute bone marrow toxicity, and incidental co-dominant C1q and IgG glomerular deposition. Creatinine level improved after treatment with prednisolone
Alsara et al. [57]	Not specified^a^	Case report	2017	1	Pregnant PNH patient	Eculizumab was started in a 30-year-old pregnant PNH patient. During ongoing pregnancy she developed infection-induced transfusion-dependent breakthrough hemolysis accompanied by acute Budd-Chiari syndrome. Treatment with anticoagulation and intensified eculizumab was tolerated and a C-section was planned
Jarrah et al. [58]	Lebanon	Case report	2022	1	PNH after COVID-19 vaccination	29-year-old female developed fatigue and shortness of breath 1 week after receiving her first mRNA COVID-19 vaccination. She was found to have pancytopenia with intravascular hemolysis, and further work-up established diagnosis of PNH

^a^Middle Eastern patient treated in Germany

FLAER, fluorescein-labeled proaerolysin; HSCT, hematopoietic stem-cell transplant; IHC, immunohistochemical staining; IUFD, intrauterine fetal death; MDS, myelodysplastic syndrome; PNH, paroxysmal nocturnal hemoglobinuria; RBC, red blood cell; SLE, systemic lupus erythematosus

Five publications originate from Saudi Arabia [45, 47, 48, 51, 56], four from Iran [46, 50, 52, 53], two from Kuwait [54, 55], and one each from Egypt [44], Oman [49], and Lebanon [58]; one article reports a Middle Eastern woman who was treated in Germany [57].

Two articles involved children only [44, 52], one involved children and adults [48], and the remainder involved adults [45–47, 49–51, 53–58]. The reports described 26 children and 209 adults in total.

### Prevalence/incidence

Only one study provided information about the epidemiology of PNH in a Middle Eastern country. A single-center study from Oman found PNH clones in 10 (7%) of 140 patients screened for PNH by flow cytometry between 2012 and 2019. From this, the authors estimated the average incidence of PNH in the Omani population to be 1.9 (range 1–3.5) per 5 million population (or 0.38 per million population/year) [49].

### Diagnosis

Five publications described the flow cytometry procedures used to screen for PNH and reported PNH clone positivity rates among patients/samples referred for PNH diagnosis. A small Egyptian study found that, among 11 newly diagnosed treatment-naïve pediatric patients with aplastic anemia who were screened for PNH by immunohistochemical bone marrow staining using CD59 monoclonal antibody, four (36%) had PNH clones [44]. A specialist Saudi Arabian center performed multiparametric flow cytometry analysis using the markers CD235a and CD59 on red blood cells, and FLAER and antibodies to CD14, CD45, CD64, CD24, and CD15 on granulocytes and monocytes. Among 366 peripheral blood samples sent for PNH screening (2012–2013), 14 (4%) were positive for PNH clones [45]. In a more recent report, 4% of 183 Saudi Arabian patients with severe persistent cytopenia(s) referred to a reference center (January 2009 to March 2016) for bone marrow evaluation to rule out myelodysplastic syndrome were found to have PNH [48]. An Iranian center (2014–2019) set up standardized multicolor flow cytometry assays (initially 3-color, but later 4-color) using CD235a, CD59, and CD55 for red blood cell immunophenotyping, FLAER, CD45, CD15, and CD24 for granulocyte immunophenotyping, and FLAER, CD45, CD46, and CD14 for monocyte immunophenotyping. Among 671 patients referred for laboratory assessment of possible PNH, including flow cytometry, 81 (12.1%) were diagnosed with PNH [50]. An Omani reference center introduced FLAER-based flow cytometry for the diagnosis of PNH in 2015, using CD55/59 assessment of neutrophils, monocytes, and red blood cells. Between 2012 and 2019, 10 (7%) of 140 patients screened for PNH using flow cytometry were diagnosed with PNH or were on follow-up for PNH [49].

### Clinical characteristics

Among 11 PNH patients aged between 18 and 50 years in a Saudi Arabian study, eight (73%) presented with aplastic anemia, and one patient each presented with pancytopenia, chronic immune thrombocytopenia, or Budd-Chiari syndrome. Two of the patients with aplastic anemia also had evidence of thrombosis. The median clone size was 15% in red blood cells (CD59) and 63% in granulocytes and monocytes (FLAER/CD24) [45].

In an Omani case series (*n* = 10), the median age at diagnosis was 22.5 (range 16–49) years, and the most common symptoms were anemia and fatigue. Six patients had classical PNH with hemolytic anemia and large PNH clones (> 60% neutrophils [FLAER/CD24] and monocytes [FLAER/CD14), three had PNH with small clones (< 10% granulocytes and monocytes) in the context of another bone marrow disorder, and one had subclinical PNH with a clone size < 1% [49].

A retrospective review of 81 patients with PNH in Iran found that the mean age at diagnosis was 38 (range 11–79) years. Among 59 patients with available information about clinical manifestations, 38 (64%) had a history of anemia, and 13 (22%) had previously presented with aplastic anemia. Other reported symptoms were fatigue, jaundice, hematuria, abdominal pain, and bruising. At the time of diagnosis, 91% (74/81) of patients had anemia, and 50% had pancytopenia. The average clone size was 30% in red blood cells (CD55/59) and 59% in granulocytes (FLAER/CD45/15/24) and monocytes (FLAER/CD45/64/14). On average, PNH clone sizes were largest in the age group 25–45 years across all cell lineages (red blood cells, granulocytes, and monocytes) [50].

### Treatment

In a series of ten patients with PNH in Oman between 2012 and 2019, five were treated with eculizumab. Two patients, one with classical PNH and one with PNH associated with another bone marrow disorder, showed a good response. In contrast, two other patients, both with classical PNH, had symptomatic improvement with eculizumab but continued to experience mild hemolysis, which was managed with intermittent transfusions. The response to eculizumab was not specified for one patient with classical PNH who subsequently underwent bone marrow transplantation [49].

A case report from Kuwait described the use of eculizumab without anticoagulant therapy in a high-risk patient presenting PNH complicated by Budd-Chiari syndrome thrombosis and a hepatic portal vein stent. PNH was managed initially with blood transfusions and anticoagulants. Following deterioration of her condition, the patient commenced treatment with eculizumab which resulted in rapid improvement. Despite the high risk of thrombosis due to Budd-Chiari syndrome and the placed stent, the patient declined anticoagulation since starting eculizumab. More than four years later, no further thrombotic events had occurred during use of eculizumab without anticoagulation [55].

Case reports reported between 1998 and 2000, which was from the pre-eculizumab era, described the reversal of acute renal failure in PNH patients in Saudi Arabia and Iran. Two patients who presented with acute renal failure were diagnosed with PNH. In two other cases, acute renal failure developed in patients with known PNH, one with concurrent aplastic anemia. All patients responded to hemodialysis, with a return to normal renal function after 2–10 weeks [51–53].

A retrospective single-center study from Iran reported outcomes in 13 patients with PNH who underwent allogeneic hematopoietic stem cell transplantations between 2002 and 2014. The mean age of patients at the time of the transplant was 27.5 years. Three patients died, all due to graft-versus-host disease. The 13-year survival rate was 74%. A significant relationship was identified between a history of thrombosis and increased mortality [46]. In a study from Oman, two of four patients (out of ten patients in total with PNH) who underwent bone marrow transplantations developed chronic graft-versus-host disease [49].

### Special patient populations

#### Pregnancy

Pregnancy in patients with PNH is known to increase the risk of maternal and fetal morbidity and mortality [3]. A case series from Saudi Arabia described ten pregnancies among four women with known PNH. The patients received transfusions as required during the antepartum period, and all received anticoagulants during both the antepartum and postpartum periods. Three of the women received eculizumab during the antepartum and postpartum periods; one did not but the reason was not documented. Eculizumab dosage and frequency tended to increase during pregnancy. Two pregnancies had planned induction of labor, two required cesarean section, two ended in spontaneous abortion, two in missed abortion requiring evacuation, one required termination following intrauterine fetal death, and one pregnancy was ongoing at the time of reporting. Three of the four live-born infants were healthy, and one was born with congenital anomalies (of which there was a family history). One woman developed a mesenteric vein thrombosis in the postpartum period [47].

A case report from Germany described a Middle Eastern patient with PNH previously managed with blood transfusions who developed transfusion-dependent breakthrough hemolysis during pregnancy. Eculizumab was initiated, and her condition improved. Two months later, she was diagnosed with Budd-Chiari syndrome requiring full anticoagulation alongside eculizumab intensification. The pregnancy was continued, and she underwent planned cesarean section [57].

#### Unusual presentations and comorbidities

A 60-year-old Kuwaiti patient with systemic lupus erythematosus developed portal vein thrombosis and thrombocytopenia and was subsequently diagnosed with PNH [54]. A patient in Saudi Arabia was diagnosed with PNH during assessment of anemia and acute kidney injury that developed after she had consumed herbal medicine of unknown constituents. The clinical picture included acute bone marrow toxicity, severe renal hemosiderosis, acute interstitial nephritis, probably herb-induced, and incidental co-dominant glomerular C1q deposition, as well as previously undiagnosed PNH. It was hypothesized that the PNH-associated reduction in CD59 might have led to a reduction in calreticulin (which acts as a C1q receptor on neutrophils), leading to an excess of circulating unbound C1q and glomerular C1q deposition [56]. A young woman from Lebanon developed pancytopenia after receiving her first dose of mRNA COVID-19 vaccination and was subsequently diagnosed with PNH, suggesting that COVID-19 vaccination may trigger exacerbation of underlying (unknown) PNH in some individuals [58].

## Discussion

The systematic literature search conducted for this review retrieved a limited number of published reports about PNH in the Middle East, mostly from Saudi Arabia and Iran, thus restricting our ability to draw definitive conclusions. Notwithstanding, as this is the first publication to our knowledge to collect and report data on PNH specific to the Middle East, it has value in terms of providing initial insight into the clinical characteristics and management of PNH in the region.

The estimated incidence rate of PNH (0.38 cases per 1 million individuals/year) reported in an Omani cohort [49] was lower than that reported in the UK (3.5 cases per 1 million individuals/year) [11], although no conclusions can be drawn based on the results of one study from a single country within the region. Importantly, population-based registries are lacking in the Middle East and underdiagnosis of the disorder is likely. Variable innate factors related to PNH contribute to its rare diagnosis and may have compromised the effective implementation of consolidative and robust diagnostic strategies for potential patients with PNH in the Middle East. Challenges to timely access to PNH confirmatory studies can bias the true prevalence of this condition. In fact, patients who were specifically screened for PNH using flow cytometry had rates of confirmed PNH that ranged from 4 to 12% [45, 48–50]. Aligning with expert consensus that regards peripheral blood flow cytometry as the gold standard test for PNH diagnosis [25], this technique was utilized in Saudi Arabia [45, 48], Oman [49] and Iran [50].

The clinical characteristics of Middle Eastern patients reported in the retrieved articles were consistent with the known disease profile of PNH [2, 3, 8]. Patients’ ages at diagnosis reported in the Middle East [49, 50] were broadly consistent with median and mean ages of 35.5 and 39.3 years, respectively, reported in an International PNH Registry analysis [8]. Common symptoms reported in Middle Eastern patients with PNH were anemia and fatigue [45, 49, 50], as also described in the International PNH Registry patient population [8].

C5 inhibitors are considered standard-of-care treatments for patients with classical PNH [16]. Ravulizumab is increasingly used because of its 8-weekly schedule and favorable breakthrough-hemolysis profile [28–31]. However, uptake of ravulizumab in the Middle East remains undocumented: few of the available Middle Eastern publications reported on use of eculizumab, and none described use of ravulizumab (possibly due to its more recent introduction). Where eculizumab use was reported, it generally improved patients’ condition [47, 49, 55, 57], which aligns with its reported efficacy in PNH [26, 27]. In some countries, barriers to treatment access may be present, such as delays in regulatory approval of therapies, the high cost of drugs, and the need for meningococcal vaccination in patients with PNH.

Acute renal impairment can occur in patients with PNH. C5 inhibition improves renal function, although dialysis may still be necessary in some patients [59, 60]. Case reports from the Middle East, published before the introduction of eculizumab, indicated that patients with PNH-associated acute renal failure responded well to hemodialysis, with a return to normal renal function [51–53].

Pregnancy in women with PNH is associated with an increased risk of morbidity and mortality [1, 3]. Observational studies suggest that eculizumab can be administered during pregnancy without adverse maternal or fetal consequences [61], although a dose increase may be required to maintain effective control of hemolysis [62]. In a review of 37 pregnancies with eculizumab exposure in the UK, 84% resulted in live births, and there were no reported cases of maternal thrombosis and no maternal or fetal deaths [62]. A case series of ten pregnancies in four Saudi Arabian women found a general increase in the eculizumab dose during pregnancy. Although, proportionally, there were more adverse pregnancy outcomes in the Saudi Arabian series than in the UK study, the Saudi Arabian series was small and eculizumab was used in only seven of the ten pregnancies (in three women) [47], thus precluding any conclusions. More recently, a retrospective case series from Germany analyzed six pregnancies in five women receiving ravulizumab. All six pregnancies resulted in the birth of healthy children with normal development during a median follow-up of 13 months. Ravulizumab maintained control of hemolysis during pregnancy despite it being a recognized complement-activating condition [63].

The review is limited by publication type (retrospective studies, case series, case reports) and their inherent risk of bias, and by the small number of available published reports on PNH patients in the Middle East, although this is not unusual for rare diseases. Clinical data were limited overall. Only a few publications described the use of eculizumab and none reported on use of newer agents including ravulizumab. Owing to the relative scarcity and age of PNH literature from the Middle East (53% of articles were published before 2016), it is possible that the range of studies identified for review does not accurately reflect current management of the condition in the region. Consequently, the available data must be interpreted cautiously.

In conclusion, and subject to the abovementioned limitations, the available published information on PNH in the Middle East suggests that the disease presentation and management approach are generally consistent with that in populations outside the region. However, additional data are required to gain a clearer picture of the status of PNH and its management in the Middle East region. Enrolling patients from Middle Eastern countries in international registries may be a useful first step toward achieving this goal.

## Data Availability

No datasets were generated or analysed during the current study.

## References

[1] Parker C, Omine M, Richards S, et al. Diagnosis and management of paroxysmal nocturnal hemoglobinuria. Blood. 2005;106(12):3699–709. 10.1182/blood-2005-04-1717. 16051736 10.1182/blood-2005-04-1717 PMC1895106

[2] Brodsky RA. Paroxysmal nocturnal hemoglobinuria. Blood. 2014;124(18):2804–11. 10.1182/blood-2014-02-522128. 25237200 10.1182/blood-2014-02-522128 PMC4215311

[3] Hill A, DeZern AE, Kinoshita T, Brodsky RA. Paroxysmal nocturnal haemoglobinuria. Nat Rev Dis Primers. 2017;3: 17028. 10.1038/nrdp.2017.28. 28516949 10.1038/nrdp.2017.28 PMC7879566

[4] Shah N, Bhatt H. Paroxysmal nocturnal hemoglobinuria. 2023 Jul 31. In: StatPearls. Treasure Island: StatPearls Publishing; 2025. 32965963

[5] Schrezenmeier H, Muus P, Socié G, et al. Baseline characteristics and disease burden in patients in the International Paroxysmal Nocturnal Hemoglobinuria Registry. Haematologica. 2014;99(5):922–9. 10.3324/haematol.2013.093161. 24488565 10.3324/haematol.2013.093161 PMC4008114

[6] Socié G, Schrezenmeier H, Muus P, et al. Changing prognosis in paroxysmal nocturnal haemoglobinuria disease subcategories: an analysis of the International PNH Registry. Intern Med J. 2016;46(9):1044–53. 10.1111/imj.13160. 27305361 10.1111/imj.13160

[7] Almeida AM, Bedrosian C, Cole A, et al. Clinical benefit of eculizumab in patients with no transfusion history in the International Paroxysmal Nocturnal Haemoglobinuria Registry. Intern Med J. 2017;47(9):1026–34. 10.1111/imj.13523. 28608499 10.1111/imj.13523

[8] Schrezenmeier H, Röth A, Araten DJ, et al. Baseline clinical characteristics and disease burden in patients with paroxysmal nocturnal hemoglobinuria (PNH): updated analysis from the International PNH registry. Ann Hematol. 2020;99(7):1505–14. 10.1007/s00277-020-04052-z. 32390114 10.1007/s00277-020-04052-z PMC7316848

[9] Hill A, de Latour RP, Kulasekararaj AG, et al. Concomitant immunosuppressive therapy and eculizumab use in patients with paroxysmal nocturnal hemoglobinuria: an International PNH Registry analysis. Acta Haematol. 2023;146(1):1–13. 10.1159/000526979. 36108594 10.1159/000526979

[10] Jalbert JJ, Chaudhari U, Zhang H, Weyne J, Shammo JM. Epidemiology of PNH and real-world treatment patterns following an incident PNH diagnosis in the US. Blood. 2019;134(Supplement_1):3407. 10.1182/blood-2019-125867.

[11] Richards SJ, Painter D, Dickinson AJ, et al. The incidence and prevalence of patients with paroxysmal nocturnal haemoglobinuria and aplastic anaemia PNH syndrome: a retrospective analysis of the UK’s population-based haematological malignancy research network 2004–2018. Eur J Haematol. 2021;107(2):211–8. 10.1111/ejh.13640. 34060690 10.1111/ejh.13640

[12] Urbano-Ispizua Á, Muus P, Schrezenmeier H, Almeida AM, Wilson A, Ware RE. Different clinical characteristics of paroxysmal nocturnal hemoglobinuria in pediatric and adult patients. Haematologica. 2017;102(3):e76–9. 10.3324/haematol.2016.151852. 27884975 10.3324/haematol.2016.151852 PMC5394949

[13] Kulagin AD, Klimova OU, Dobronravov AV, et al. Paroxysmal nocturnal hemoglobinuria in children and adults: comparative clinical profile and long-term prognosis. Pediatric Hematol Oncol Immunopathol. 2018;17(3):11–21. 10.24287/1726-1708-2018-17-3-11-21.

[14] Shen W, Clemente MJ, Hosono N, et al. Deep sequencing reveals stepwise mutation acquisition in paroxysmal nocturnal hemoglobinuria. J Clin Invest. 2014;124(10):4529–38. 10.1172/JCI74747. 25244093 10.1172/JCI74747 PMC4191017

[15] Kinoshita T, Medof ME, Silber R, Nussenzweig V. Distribution of decay-accelerating factor in the peripheral blood of normal individuals and patients with paroxysmal nocturnal hemoglobinuria. J Exp Med. 1985;162(1):75–92. 10.1084/jem.162.1.75. 2409211 10.1084/jem.162.1.75 PMC2187705

[16] Risitano AM, Frieri C, Urciuoli E, Marano L. The complement alternative pathway in paroxysmal nocturnal hemoglobinuria: from a pathogenic mechanism to a therapeutic target. Immunol Rev. 2023;313(1):262–78. 10.1111/imr.13137. 36110036 10.1111/imr.13137 PMC10087358

[17] Rother RP, Bell L, Hillmen P, Gladwin MT. The clinical sequelae of intravascular hemolysis and extracellular plasma hemoglobin: a novel mechanism of human disease. JAMA. 2005;293(13):1653–62. 10.1001/jama.293.13.1653. 15811985 10.1001/jama.293.13.1653

[18] Xie CB, Jane-Wit D, Pober JS. Complement membrane attack complex: new roles, mechanisms of action, and therapeutic targets. Am J Pathol. 2020;190(6):1138–50. 10.1016/j.ajpath.2020.02.006. 32194049 10.1016/j.ajpath.2020.02.006 PMC7280757

[19] Waheed A, Shammo J, Dingli D. Paroxysmal nocturnal hemoglobinuria: review of the patient experience and treatment landscape. Blood Rev. 2024;64: 101158. 10.1016/j.blre.2023.101158. 38071133 10.1016/j.blre.2023.101158

[20] Lee JW, Jang JH, Kim JS, et al. Clinical signs and symptoms associated with increased risk for thrombosis in patients with paroxysmal nocturnal hemoglobinuria from a Korean registry. Int J Hematol. 2013;97(6):749–57. 10.1007/s12185-013-1346-4. 23636668 10.1007/s12185-013-1346-4

[21] Jang JH, Kim JS, Yoon SS, et al. Predictive factors of mortality in population of patients with paroxysmal nocturnal hemoglobinuria (PNH): results from a Korean PNH registry. J Korean Med Sci. 2016;31(2):214–21. 10.3346/jkms.2016.31.2.214. 26839475 10.3346/jkms.2016.31.2.214 PMC4729501

[22] Brodsky RA, Lee JW, Nishimura JI, Szer J. Lactate dehydrogenase versus haemoglobin: which one is the better marker in paroxysmal nocturnal haemoglobinuria? Br J Haematol. 2022;196(2):264–5. 10.1111/bjh.17860. 34923628 10.1111/bjh.17860

[23] Röth A, Maciejewski J, Nishimura JI, Jain D, Weitz JI. Screening and diagnostic clinical algorithm for paroxysmal nocturnal hemoglobinuria: expert consensus. Eur J Haematol. 2018;101(1):3–11. 10.1111/ejh.13059. 29532535 10.1111/ejh.13059

[24] Cançado RD, Araújo ADS, Sandes AF, et al. Consensus statement for diagnosis and treatment of paroxysmal nocturnal haemoglobinuria. Hematol Transfus Cell Ther. 2021;43(3):341–8. 10.1016/j.htct.2020.06.006. 32713742 10.1016/j.htct.2020.06.006 PMC8446255

[25] McKeage K. Eculizumab: a review of its use in paroxysmal nocturnal haemoglobinuria. Drugs. 2011;71(17):2327–45. 10.2165/11208300-000000000-00000. 22085388 10.2165/11208300-000000000-00000

[26] Hillmen P, Muus P, Dührsen U, et al. Effect of the complement inhibitor eculizumab on thromboembolism in patients with paroxysmal nocturnal hemoglobinuria. Blood. 2007;110(12):4123–8. 10.1182/blood-2007-06-095646. 17702897 10.1182/blood-2007-06-095646

[27] Zhou S, Dong X, Chen C, et al. Efficacy and safety of eculizumab for paroxysmal nocturnal hemoglobinuria: a systematic review and meta-analysis. J Pediatr Hematol Oncol. 2021;43(6):203–10. 10.1097/MPH.0000000000002178. 33902068 10.1097/MPH.0000000000002178

[28] Lee JW, Sicre de Fontbrune F, Wong Lee Lee L, et al. Ravulizumab (ALXN1210) vs eculizumab in adult patients with PNH naive to complement inhibitors: the 301 study. Blood. 2019;133(6):530–9. 10.1182/blood-2018-09-876136. 30510080 10.1182/blood-2018-09-876136 PMC6367644

[29] Kulasekararaj AG, Hill A, Rottinghaus ST, et al. Ravulizumab (ALXN1210) vs eculizumab in C5-inhibitor-experienced adult patients with PNH: the 302 study. Blood. 2019;133(6):540–9. 10.1182/blood-2018-09-876805. 30510079 10.1182/blood-2018-09-876805 PMC6368201

[30] Lee JW, Kulasekararaj AG. Ravulizumab for the treatment of paroxysmal nocturnal hemoglobinuria. Expert Opin Biol Ther. 2020;20(3):227–37. 10.1080/14712598.2020.1725468. 32011183 10.1080/14712598.2020.1725468

[31] Ishiyama K, Usuki K, Ikezoe T, et al. Japanese patient preferences between ravulizumab and eculizumab for the treatment of paroxysmal nocturnal hemoglobinuria. Rinsho Ketsueki. 2023;64(1):9–17. 10.11406/rinketsu.64.9. (**in Japanese**). 36775313 10.11406/rinketsu.64.9

[32] Rehan ST, Hashmi MR, Asghar MS, Tahir MJ, Yousaf Z. Pegcetacoplan - a novel C3 inhibitor for paroxysmal nocturnal hemoglobinuria. Health Sci Rep. 2022;5(3): e512. 10.1002/hsr2.512. 35509414 10.1002/hsr2.512 PMC9059189

[33] Heo YA. Pegcetacoplan: a review in paroxysmal nocturnal haemoglobinuria. Drugs. 2022;82(18):1727–35. 10.1007/s40265-022-01809-w.Erratum.In:Drugs2023Jun1. 36459381 10.1007/s40265-022-01809-w PMC10234880

[34] Hillmen P, Szer J, Weitz I, et al. Pegcetacoplan versus eculizumab in paroxysmal nocturnal hemoglobinuria. N Engl J Med. 2021;384(11):1028–37. 10.1056/NEJMoa2029073. 33730455 10.1056/NEJMoa2029073

[35] de Latour RP, Szer J, Weitz IC, et al. Pegcetacoplan versus eculizumab in patients with paroxysmal nocturnal haemoglobinuria (PEGASUS): 48-week follow-up of a randomised, open-label, phase 3, active-comparator, controlled trial. Lancet Haematol. 2022;9(9):e648–59. 10.1016/S2352-3026(22)00210-1. 36055332 10.1016/S2352-3026(22)00210-1

[36] Lee JW, Griffin M, Kim JS, et al. Addition of danicopan to ravulizumab or eculizumab in patients with paroxysmal nocturnal haemoglobinuria and clinically significant extravascular haemolysis (ALPHA): a double-blind, randomised, phase 3 trial. Lancet Haematol. 2023;10(12):e955–65. 10.1016/S2352-3026(23)00315-0. 38030318 10.1016/S2352-3026(23)00315-0

[37] Gerber GF, Brodsky RA. Pegcetacoplan for paroxysmal nocturnal hemoglobinuria. Blood. 2022;139(23):3361–5. 10.1182/blood.2021014868. 35349667 10.1182/blood.2021014868

[38] Notaro R, Luzzatto L. Breakthrough hemolysis in PNH with proximal or terminal complement inhibition. N Engl J Med. 2022;387(2):160–6. 10.1056/NEJMra2201664. 35830642 10.1056/NEJMra2201664

[39] Peffault de Latour R, Röth A, Kulasekararaj AG, et al. Oral iptacopan monotherapy in paroxysmal nocturnal hemoglobinuria. N Engl J Med. 2024;390(11):994–1008. 10.1056/NEJMoa2308695. 38477987 10.1056/NEJMoa2308695

[40] Syed YY. Iptacopan: first approval. Drugs. 2024;84(5):599–606. 10.1007/s40265-024-02009-4. 38517653 10.1007/s40265-024-02009-4

[41] Risitano AM, Kulasekararaj AG, Lee JW, et al. Danicopan: an oral complement factor D inhibitor for paroxysmal nocturnal hemoglobinuria. Haematologica. 2021;106(12):3188–97. 10.3324/haematol.2020.261826. 33121236 10.3324/haematol.2020.261826 PMC8634185

[42] Kang C. Danicopan: first approval. Drugs. 2024;84(5):613–8. 10.1007/s40265-024-02023-6. 38528310 10.1007/s40265-024-02023-6

[43] Peffault de Latour R, Schrezenmeier H, Bacigalupo A, et al. Allogeneic stem cell transplantation in paroxysmal nocturnal hemoglobinuria. Haematologica. 2012;97(11):1666–73. 10.3324/haematol.2012.062828. 22689687 10.3324/haematol.2012.062828 PMC3487438

[44] Rizk S, Ibrahim IY, Mansour IM, Kandil D. Screening for paroxysmal nocturnal hemoglobinuria (PNH) clone in Egyptian children with aplastic anemia. J Trop Pediatr. 2002;48(3):132–7. 10.1093/tropej/48.3.132. 12164595 10.1093/tropej/48.3.132

[45] AlGhasham N, Abulkhair Y, Khalil S. Flow cytometry screening for paroxysmal nocturnal hemoglobinuria: A single-center experience in Saudi Arabia. Cytometry B Clin Cytom. 2015;88(6):389–94. 10.1002/cyto.b.21317. 26296648 10.1002/cyto.b.21317

[46] Kamranzadeh Fumani H, Zokaasadi M, Kasaeian A, et al. Allogeneic hematopoietic stem cell transplantation for paroxysmal nocturnal hemoglobinuria: a retrospective single-center study. Hematol Oncol. 2017;35(4):935–8. 10.1002/hon.2367. 27761934 10.1002/hon.2367

[47] Al-Dosari YM, Al-Zahrani H, Al-Mohareb F, Hashmi S. Pregnancy with paroxysmal nocturnal hemoglobinuria: a case series with review of the literature. Saudi J Med Med Sci. 2021;9(2):178–89. 10.4103/sjmms.sjmms_4_20. 34084110 10.4103/sjmms.sjmms_4_20 PMC8152383

[48] AlMozain N, Mashi A, Alneami Q, et al. Spectrum of myelodysplastic syndrome in patients evaluated for cytopenia(s). A report from a reference centre in Saudi Arabia. Hematol Oncol Stem Cell Ther. 2022;15(2):39–44. 10.1016/j.hemonc.2020.11.001. 33227261 10.1016/j.hemonc.2020.11.001

[49] Al-Riyami AZ, Al-Kindi Y, Al-Qassabi J, et al. Clinicopathological profile of paroxysmal nocturnal hemoglobinuria among Omani patients: a case series. Oman Med J. 2022;37(4): e405. 10.5001/omj.2022.13. 35949713 10.5001/omj.2022.13 PMC9357272

[50] Jahangirpour M, Vahedi A, Baghdadi H, et al. Paroxysmal nocturnal haemoglobinuria, diagnosis and haematological findings, first report from Iran, model for developing countries. EJHaem. 2022;3(2):335–40. 10.1002/jha2.410. 35846057 10.1002/jha2.410 PMC9176096

[51] Al-Harbi A, Alfurayh O, Sobh M, Akhtar M, Tashkandy MA, Shaaban A. Paroxysmal nocturnal hemoglobinuria and renal failure. Saudi J Kidney Dis Transpl. 1998;9(2):147–51. 18408291

[52] Mooraki A, Boroumand B, Mohammad Zadeh F, Ahmed SH, Bastani B. Acute reversible renal failure in a patient with paroxysmal nocturnal hemoglobinuria. Clin Nephrol. 1998;50(4):255–7. 9799072

[53] Khajehdehi P. Reversible acute renal failure with prolonged oliguria and gross hematuria in a case of paroxysmal nocturnal hemoglobinuria. Scand J Urol Nephrol. 2000;134(4):284–6. 10.1080/003655900750042068. 10.1080/003655900750042068 11095091

[54] Gupta A, Al Fulaij R, Gupta RK, Gupta G, Marouf R. Development of paroxysmal nocturnal haemoglobinuria in systemic lupus erythematosus: an unusual cause of portal vein thrombosis. Lupus. 2009;18(8):743–6. 10.1177/0961203308100558. 19502272 10.1177/0961203308100558

[55] Al-Jafar HA, AlDallal SM, Askar HA, Aljeraiwi AM, Al-Alansari A. Long standing eculizumab treatment without anticoagulant therapy in high-risk thrombogenic paroxysmal nocturnal hemoglobinuria. Hematol Rep. 2015;7(3): 5927. 10.4081/hr.2015.5927. 26487933 10.4081/hr.2015.5927 PMC4591499

[56] Boqari DT, Al Faraj S, Arafah M, et al. Herb-induced acute bone marrow intoxication and interstitial nephritis superimposing glomerular C1q deposition in a patient with paroxysmal nocturnal hemoglobinuria. Saudi J Kidney Dis Transpl. 2015;26(3):572–9. 10.4103/1319-2442.157384. 26022031 10.4103/1319-2442.157384

[57] Alsara M, Alashkar F, Baum J, et al. Transfusion-dependent breakthrough hemolysis accompanied by an acute Budd-Chiari Syndrome (BCS) in a pregnant patient with paroxysmal nocturnal hemoglobinuria (PNH) on eculizumab. Oncol Res Treat. 2017;40(Supplement 3):157. 10.1159/000479566.

[58] Jarrah K, Al Mahmasani L, Atoui A, Bou-Fakhredin R, Taher AT. Manifestation of paroxysmal nocturnal hemoglobinuria after COVID-19 mRNA vaccination. Blood Cells Mol Dis. 2022;93: 102641. 10.1016/j.bcmd.2021.102641. 34980554 10.1016/j.bcmd.2021.102641 PMC8716432

[59] Macedo ÊS, Parente Filho SLA, Pro JDZ, et al. Renal involvement in paroxysmal nocturnal haemoglobinuria: a brief review of the literature. Rev Assoc Med Bras. 2018;64(12):1139–46. 10.1590/1806-9282.64.12.1139. 30569992 10.1590/1806-9282.64.12.1139

[60] Gembillo G, Siligato R, Cernaro V, Santoro D. Complement inhibition therapy and dialytic strategies in paroxysmal nocturnal hemoglobinuria: the nephrologist’s opinion. J Clin Med. 2020;9(5): 1261. 10.3390/jcm9051261. 32357555 10.3390/jcm9051261 PMC7287718

[61] Socié G, Caby-Tosi MP, Marantz JL, et al. Eculizumab in paroxysmal nocturnal haemoglobinuria and atypical haemolytic uraemic syndrome: 10-year pharmacovigilance analysis. Br J Haematol. 2019;185(2):297–310. 10.1111/bjh.15790. 30768680 10.1111/bjh.15790 PMC6594003

[62] Manning JE, Anderson RM, Hill A, Zeidan D, Ciantar E. Pregnancy outcomes in women receiving eculizumab for the management of paroxysmal nocturnal haemoglobinuria. Obstet Med. 2022;15(1):45–9. 10.1177/1753495X211019899. 35444730 10.1177/1753495X211019899 PMC9014543

[63] Hoechsmann B, Leopold W, Schneider A, Koerper S, Kalhammer U, Schrezenmeier H. Ravulizumab in pregnant women with paroxysmal nocturnal hemoglobinuria (PNH) − favourable experience from a retrospective case series. Blood. 2024;144(Supplement 1):5251. 10.1182/blood-2024-209857.

